# Effect of Hazardous Alcohol Use During Pregnancy on Growth Outcomes at Birth: Findings from a South African Cohort Study

**DOI:** 10.1111/acer.13566

**Published:** 2017-12-28

**Authors:** Bronwyn Myers, Nastassja Koen, Kirsten A. Donald, Raymond T. Nhapi, Lesley Workman, Whitney Barnett, Nadia Hoffman, Sheri Koopowitz, Heather J. Zar, Dan J. Stein

**Affiliations:** ^1^ Alcohol, Tobacco and Other Drug Research Unit South African Medical Research Council Cape Town South Africa; ^2^ Department of Psychiatry and Mental Health University of Cape Town Cape Town South Africa; ^3^ Department of Paediatrics and Child Health Red Cross War Memorial Children's Hospital University of Cape Town Cape Town South Africa; ^4^ Division of Epidemiology and Biostatistics School of Public Health and Family Medicine University of Cape Town Cape Town South Africa; ^5^ South African Medical Research Council Unit on Child and Adolescent Health Cape Town South Africa; ^6^ South African Medical Research Council Unit on Anxiety and Stress Disorders Cape Town South Africa

**Keywords:** Antenatal Alcohol Exposure, Infant Growth Outcomes, South Africa, Cohort

## Abstract

**Background:**

Cohort studies have noted associations between hazardous alcohol use during pregnancy and infant growth outcomes, but many have not controlled for potential psychosocial confounders. To assess the unique contribution of hazardous alcohol use, we examined its effect on infant growth outcomes while controlling for maternal psychosocial stressors and hazardous tobacco and drug use in a cohort of 986 pregnant South African women enrolled into the Drakenstein Child Health Study between 2012 and 2015.

**Methods:**

Data on psychosocial stressors and maternal risk behaviors were collected between 28 and 32 weeks of gestation. Participants were categorized as hazardous alcohol users if they obtained moderate or high scores (>10) on the Alcohol, Smoking and Substance Involvement Screening Test at this assessment or retrospectively reported drinking at least 2 drinks weekly during any trimester of pregnancy. Infant growth outcomes were recorded at delivery. Multivariable regression models examined correlates of hazardous alcohol use and associations between hazardous alcohol use and birth outcomes.

**Results:**

Overall, 13% of mothers reported hazardous alcohol use. Recent exposure to intimate partner violence (adjusted odds ratio (aOR) = 2.08; 95% confidence interval (CI): 1.37, 3.18) and hazardous tobacco use (aOR = 5.03; 95% CI: 2.97, 8.52) were significant correlates of hazardous alcohol use. After controlling for potential psychosocial confounders, hazardous alcohol use remained associated with lower infant weight‐for‐age (*B* = −0.35, 95% CI: −0.56, −0.14), height‐for‐age (*B* = −0.46, 95% CI: −0.76, −0.17), and head‐circumference‐for‐age *z*‐scores (*B* = −0.43, 95% CI: −0.69, −0.17).

**Conclusions:**

Interventions to reduce hazardous alcohol use among pregnant women in South Africa are needed to prevent alcohol‐related infant growth restrictions. As these growth deficits may lead to neurodevelopmental consequences, it is critical to identify alcohol‐related growth restrictions at birth and link exposed infants to early interventions for neurodevelopment.

Relative to other countries, South Africa has one of the highest levels of alcohol consumption and heavy episodic drinking per drinker (World Health Organization, 2014). The Western Cape is particularly affected by alcohol use disorders, with a nationally representative study estimating that 20% of adults in this province (compared to a national average of 13%) were likely to develop a substance use disorder in their lifetime—with alcohol being the most commonly used substance (Stein et al., 2008).

Although many women in the Western Cape decrease their alcohol consumption after pregnancy recognition, a significant proportion continue to consume alcohol at levels that increase risk of health and social harms, referred to as hazardous drinking (Choi et al., 2014; O'Connor et al., 2011; Williams et al., 2014). Findings from a representative survey of 5,231 pregnant women receiving antenatal care at 11 midwife‐run obstetric units confirm the high prevalence of recent alcohol use among pregnant women in this region, with 20% of the sample testing positive for past‐week alcohol use (Williams et al., 2014).

These findings are cause for concern, given the consequences of hazardous alcohol exposure for fetal development. Fetal alcohol spectrum disorders (FASDs), a set of severe and irreversible developmental disorders characterized by growth retardation and neurodevelopmental disabilities (Kodituwakku, 2009; May et al., 2005, 2008), are the most well‐documented consequence of high concentrations of alcohol exposure in utero. A recent systematic review found that of 187 countries, South Africa has the highest prevalence of FASD, estimated at 111.1 per 1,000 population (95% CI: 71.1 to 158.4 per 1,000 population; Lange et al., 2017). Other adverse infant outcomes associated with hazardous alcohol exposure include preterm birth, low birthweight, intrauterine growth restriction, being small for gestational age (SGA), and having low weight for height and head circumferences (Mullally et al., 2011; Patra et al., 2011; Pfinder et al., 2013). These growth restrictions have implications for neurocognitive development (Arcangeli et al., 2012; Carter et al., 2016) and appear to persist into adulthood (Carter et al., 2013).

Consequently, there is a public health imperative to reduce hazardous alcohol use among pregnant women in the Western Cape and ensure the early detection and linkage to care of infants exposed to hazardous alcohol use. To facilitate early detection of alcohol‐exposed infants, a better understanding of the relationship between hazardous alcohol use during pregnancy and birth outcomes in the context of other maternal psychosocial stressors and risk behaviors is required. A handful of cohort studies have examined associations between maternal physical health (such as nutritional status and pregnancy weight), maternal risk behaviors (such as tobacco and illicit drug use), hazardous alcohol use, and birth outcomes among South African populations (e.g., Carter et al., 2007, 2012). While valuable, these earlier studies did not control for the possible effects of other maternal psychosocial stressors on birth outcomes. As maternal depression, traumatic experiences, stress, and exposure to intimate partner violence (IPV) are direct risks for adverse infant birth outcomes (Grote et al., 2010; Koen et al., 2014, 2016), this omission limits our understanding of the unique contribution of hazardous alcohol use to birth outcomes. A recent study of HIV‐infected women found that after controlling for a range of maternal psychosocial stressors, hazardous alcohol use during pregnancy increased the risk of infants being SGA (Sania et al., 2017). However, this cohort was limited to HIV‐infected women and did not control for maternal tobacco and illicit drug use, further risks for adverse birth outcomes (Vanker et al., 2016; Vythilingum et al., 2012). Given the limitations of previous studies, we require additional cohort studies that examine the impact of hazardous alcohol use on birth outcomes while controlling for maternal psychosocial stressors *and* other risk behaviors.

This study aimed to partially address this gap by (i) identifying correlates of hazardous alcohol use during pregnancy and (ii) examining whether hazardous alcohol use is associated with infant growth outcomes while controlling for the effect of maternal psychosocial stressors and other risk behaviors in a cohort of pregnant women from the Drakenstein Health district in the Western Cape.

## Materials and Methods

This study presents data from the Drakenstein Child Health Study (DCHS), a longitudinal birth cohort study that examines biological, environmental, and psychosocial determinants of maternal, paternal, and child health within the Drakenstein subdistrict of the Western Cape Province, South Africa (Stein et al., 2015). The Faculty of Health Sciences' Human Research Ethics Committee at the University of Cape Town (401/2009), Stellenbosch University (N12/02/0002), and the Western Cape Provincial Health Research Committee (2011RP45) approved this study.

### Setting

Drakenstein is a periurban area located about 60 km from Cape Town. It is a low‐socioeconomic region with a high prevalence of risk factors for poor maternal and infant health outcomes (Stein et al., 2015). As such, it is similar to many other periurban areas in South Africa and other low‐ and middle‐income countries. Most pregnant women residing in this region obtain health care from the public sector.

### Participants and Procedures

Trained fieldworkers screened pregnant women presenting for their first antenatal care visit (between 20 and 28 weeks of gestation) at TC Newman clinic (which serves a predominantly “Colored” or mixed ancestry population) or Mbekweni clinic (which serves a predominantly Black African population) for study eligibility between 2012 and 2015. The terms “Black African” and “Colored” are commonly used sociodemographic markers that are important proxies for ongoing health disparities in South Africa.

Pregnant women were eligible to participate in the DCHS if they were at least 18 years old, were accessing antenatal care at one of the aforementioned clinics, and had no intention to move out of the Drakenstein subdistrict within the forthcoming year. Eligible women were enrolled into the DCHS if they provided written informed consent to participate in the study. The DCHS recruited 1,225 pregnant women who provided sociodemographic and basic health information at their initial study visit. These participants were asked to return for a second study visit (28 to 32 weeks of gestation), at which time fieldworkers gathered information about exposure to psychosocial stressors, hazardous alcohol use, and other risk behaviors. At this appointment, they also received an antenatal ultrasound. All births occurred at a single central public hospital where clinical staff recorded key birth outcomes. At this point, 1,137 mothers were retained through birth, giving birth to 1,143 infants (including four sets of twins and 1 set of triplets). Between 2 and 6 weeks postpartum, a subset of mother–infant dyads were invited to another study visit. At this visit, fieldworkers asked mothers (*n* = 236) about their use of alcohol during their recent pregnancy. All study procedures occurred in a private room at the facility.

### Measures

#### Sociodemographic Characteristics

At study enrollment, we assessed maternal age, employment status (employed vs. not employed), educational achievement (primary, some secondary, completed secondary, or tertiary education), average household income (R1,000/month, R1,000 to R5,000/month, >R5,000/month), ancestry (African or mixed), body mass index (BMI), parity, and HIV status.

#### Hazardous Alcohol Use

We created a composite, dichotomous measure of antenatal exposure to hazardous alcohol use (exposure vs. no exposure) using data from the Alcohol, Smoking and Substance Involvement Screening Test (ASSIST; Humeniuk et al., 2008) and retrospectively collected data on hazardous alcohol use during pregnancy.

The ASSIST assesses substance use involvement for 10 substances including alcohol, tobacco, and other illicit drugs. There are 7 alcohol‐focused questions on the ASSIST. The first 2 items assess lifetime use and past 3‐month frequency of use. The next 5 items assess symptoms of alcohol‐related problems in the past 3 months, including experiencing a strong desire to use alcohol, social or financial problems associated with alcohol use, failing to fulfill roles and expectations because of alcohol use, others expressing concern about the person's drinking, and trying but failing to reduce alcohol use. A composite score is created by summing the scores on these items. Composite scores >10 indicate moderate‐to‐high levels of risk for alcohol problems (reflecting weekly or daily/almost daily alcohol use and negative consequences related to the quantity of alcohol consumed). The ASSIST has shown good reliability and has been extensively validated (Humeniuk et al., 2008), including for South African primary care settings (Mertens et al., 2009). In this study, the ASSIST was administered at the second study visit (28 to 32 weeks of gestation) to assess for moderate‐/high‐risk alcohol use during the past 3 months (corresponding roughly to week 14 to week 18 of pregnancy).

Due to suspected underreporting of alcohol use, we also examined retrospectively collected data on hazardous alcohol use (defined as 2 or more drinks of alcohol weekly during any trimester of pregnancy) for the subset of participants who completed this postpartum assessment. We used these 2 data sources to create a composite indicator of hazardous alcohol use. For this indicator, hazardous alcohol exposure was defined as an ASSIST score >10 *or* retrospective reports of hazardous alcohol use during pregnancy. With this approach, we were able to identify 131 participants with hazardous alcohol exposure: 113 participants with ASSIST scores >10 and a further 18 participants with ASSIST scores <10 who retrospectively reported hazardous alcohol use during pregnancy. We did not identify any participants who obtained ASSIST scores >10 during pregnancy who later retrospectively reported not drinking during pregnancy.

#### Other Maternal Risk Behaviors

Given the documented risks associated with tobacco use during the antenatal period (Vanker et al., 2016), we used the ASSIST to examine tobacco use during the past 3 months (week 14 to week 18 of pregnancy). For the tobacco items, ASSIST composite scores >3 indicate moderate‐to‐high levels of risk for tobacco problems (Humeniuk et al., 2008). As the data were skewed, we used this validated cutoff to categorize participants into nonhazardous versus hazardous tobacco use (where hazardous use is defined as moderate–high risk for tobacco‐related problems).

Only a small proportion of women (3%) reported any illicit drug use, of which cannabis was the most frequently reported drug used. Due to the small numbers of participants who reported drug use, we were unable to examine the effects of drug class on birth outcomes. As a result of this, and because any type of illicit drug use during pregnancy is likely to increase risk for adverse birth outcomes (Vythilingum et al., 2012), we combined the ASSIST illicit drug categories into a single “drug use” category. For the drug items, ASSIST composite scores >3 indicate moderate‐to‐high risk for drug problems (Humeniuk et al., 2008). As the data were skewed, we used this validated cutoff to categorize participants into nonhazardous versus hazardous drug use (where hazardous use is defined as moderate–high risk for drug‐related problems).

#### Maternal Psychosocial Stressors

Maternal exposure to childhood trauma was assessed using the Childhood Trauma Questionnaire—Short Form (Bernstein et al., 1994). We created groups of participants with a history of childhood trauma versus those without. The IPV Questionnaire assessed recent (past‐year) exposure to IPV. Participants were grouped into those with no exposure (score = 0) versus any exposure (score ≥1). The World Mental Health Life Events Questionnaire assessed recent exposure to stressful life events, and the median number of life events was recorded (Myer et al., 2008). The Beck Depression Inventory screened for depressive symptoms. Using a cutoff score of ≥20, we classified participants as above or below the threshold for possible depression (Lasa et al., 2000). Stein and colleagues (2015) describe these measures more fully.

#### Birth Outcomes

Birth outcomes included infant sex, birthweight (in grams), birth length, gestational age, and head circumference. Gestational age at delivery was calculated from participants' antenatal ultrasound. If this was not available, then height of fundus at enrollment or maternal recall of last menstrual period was used. Gestational age was calculated in completed weeks of gestation. Births occurring before 37 weeks of gestation were defined as preterm births. We used birthweight and gestational age to calculate the number of low‐birthweight (<2.5 kg) and SGA (defined as birthweight below the 10th percentile for babies of the same gestational age) infants. We used birthweight, birth length, gestational age, and head circumference to calculate infant weight‐for‐age (WAZ), height‐for‐age (HAZ), and head‐circumference‐for‐age (HCZ) *z*‐scores, using the revised Fenton preterm growth charts (Fenton and Kim, 2013; Fenton et al., 2013).

### Statistical Analysis

Data were analyzed using Stata 14 (StataCorp Inc, College Station, TX). We generated descriptive statistics for sociodemographic variables, psychosocial stressors, hazardous alcohol exposure, hazardous tobacco and drug use, and birth outcomes. We used chi‐square tests for categorical, Student's *t*‐tests for normally distributed, and Wilcoxon rank‐sum tests for nonnormally distributed continuous variables to assess for potential recruitment site differences. Next, we conducted logistic regression analyses to examine unadjusted and adjusted associations between hazardous tobacco and drug use, sociodemographic factors, psychosocial stressors, and hazardous alcohol use during pregnancy. All variables significantly associated (*p* < 0.05) with hazardous alcohol use in univariate analyses were entered into the multivariable model. Results are reported as odds ratios (ORs) and adjusted odds ratios (aORs) with 95% confidence intervals (CIs) for the categorical hazardous alcohol use outcome. Using simple and multiple linear regression analyses, we then modeled hazardous alcohol exposure as a predictor of infant WAZ, HAZ, and HCZ scores, resulting in 3 multiple linear regression models. All variables significantly associated with the outcome of interest (*p* < 0.05) in univariate analyses were entered into the multivariable models. The results of these regression models are reported as unstandardized regression coefficients (*B*) with 95% CIs.

## Results

### Maternal Sociodemographic Characteristics

Data on 986 mother–infant dyads who completed all study procedures are included in the analysis. The analysis excludes data from participants for whom psychosocial assessments were incomplete, for whom infant outcome data were not available, and who had multiple births. We excluded multiple births from the analysis given the small number and because of the known impact on fetal growth.

Table 1 presents maternal sociodemographic characteristics. The median age of mothers at enrollment was 26 (interquartile range [IQR] 22 to 31) years. Most participants had not completed high school (64%) and were unemployed (74%). More than a third of participants (39%) reported a household income of <R1,000 (~70 U.S. dollars) per month, and 22% were HIV‐infected. In general, mothers in this sample had adequate nutrition, with a median BMI of 27.14 (IQR: 23.63 to 32.11). Mothers recruited from Mbekweni were more likely to be older, have higher BMIs, report less household income, and be HIV‐positive than mothers recruited from TC Newman.

**Table 1:** Description of Maternal Demographic Characteristics, Psychosocial Stressors, and Infant Birth Outcomes, by Site

Variable	Mbekweni *n* (%)	TC Newman *n* (%)	Total *n* (%)	*p*‐Value
Maternal demographic characteristics (*n* = 986)				
Number of mothers	531 (53.85)	511 (46.15)	986 (100.00)	
Median age (IQR)	26.75 (22.27; 31.66)	24.82 (21.34; 29.15)	25.75 (21.96; 30.78)	<0.001
Ancestry				
African	524 (98.87)	6 (1.13)	529 (53.71)	
Mixed ancestry	5 (1.10)	450 (98.90)	456 (46.29)	<0.001
Educational attainment				
Primary	46 (8.66)	35 (7.69)	81 (8.22)	
Some secondary	292 (54.99)	236 (51.87)	528 (53.55)	
Completed secondary	157 (29.57)	164 (36.04)	321 (32.56)	
Any tertiary	36 (6.78)	20 (4.40)	56 (5.68)	0.096
Unemployed	407 (76.65)	320 (70.33)	727 (73.73)	0.025
Average household income				
<R1,000/month	235 (44.26)	147 (32.31)	382 (38.74)	
R1,000 to R5,000/month	241 (45.39)	227 (49.89)	468 (47.46)	
>R5,000/month	55 (10.36)	81 (17.80)	136 (13.79)	<0.001
HIV‐seropositive	196 (36.91)	16 (3.52)	212 (21.50)	<0.001
Median maternal BMI (IQR)	28.33 (24.61; 33.47)	25.82 (22.48; 29.84)	27.14 (23.63; 32.11)	<0.001
Parity				
1 child	204 (58.29)	152 (57.36)	356 (57.89)	
2 children	102 (29.14)	74 (27.92)	176 (28.62)	0.572
3 or more children	44 (12.57)	39 (14.72)	83 (12.77)	
Maternal psychosocial stressors (*n* = 986)				
Exposure to childhood trauma	153 (28.81)	188 (41.32)	341 (34.58)	<0.001
Median number of stressful life events (IQR)	1 (0; 2)	2 (1; 4)	1 (0; 3)	<0.001
Antenatal depression—screened positive	101 (19.02)	126 (27.69)	227 (23.02)	0.001
Recent intimate partner violence	149 (28.06)	184 (40.44)	333 (33.77)	<0.001
Hazardous alcohol use	43 (8.10)	88 (19.34)	131 (13.29)	<0.001
Hazardous tobacco use	29 (5.46)	250 (54.95)	279 (28.30)	<0.001
Hazardous drug use	3 (0.56)	29 (6.37)	32 (3.25)	<0.001
Infant birth outcomes (*n* = 986)				
Sex (female)	270 (50.85)	206 (45.27)	476 (48.28)	0.081
Median gestation at delivery (IQR)	39 (38; 40)	39 (37; 40)	39 (37; 40)	0.126
Preterm birth (<37 weeks of gestation)	119 (22.41)	118 (25.93)	237 (24.04)	0.197
Median birthweight in grams (IQR)	3170 (2830; 3455)	3000 (2630; 3350)	3090 (2720; 3410)	<0.001
Low birthweight (<2.5 kg)	58 (10.92)	87 (19.12)	145 (14.71)	0.001
Small for gestational age	122 (23.11)	127 (27.91)	249 (25.33)	0.014
Median WAZ (IQR)	−0.43 (−1.23; 0.24)	−0.71 (−1.36; −0.07)	−0.55 (−1.31; 0.07)	<0.001
Median HCZ (IQR) (*n* = 974)	−0.42 (−1.24; 0.41)	−0.61 (−1.34; 0.15)	−0.54 (−1.29; 0.15)	<0.001
Median HAZ (IQR) (*n* = 970)	0.09 (−0.82; 1.01)	−0.03 (−0.87; 0.82)	0.03 (−0.86; 0.93)	0.060

### Exposure to Psychosocial Stressors and Risk Behaviors

Of the 986 women included in the analysis, 34% reported a history of childhood trauma, 34% reported recent exposure to IPV, 23% screened positive for probable depression, 13% reported hazardous alcohol use during pregnancy, 28% reported hazardous tobacco use, and 3% reported hazardous drug use. Mothers recruited from TC Newman were significantly more likely to report hazardous alcohol, tobacco, or drug use; to report exposure to childhood trauma, stressful life events, or recent exposure to IPV; and to have probable depression than mothers recruited from Mbekweni (Table 1).

### Infant Birth Characteristics

Almost half of the infants were female (48%). The median gestational age at delivery was 39 (IQR: 37 to 40) weeks. A quarter of all births were preterm. Fifteen percent of all infants had low birthweight, and a quarter were SGA. Infants born at TC Newman were more likely to have low birthweight and be SGA than those born at Mbekweni. Overall, the median birthweight was 3,090 (IQR: 2,720 to 3,410) grams. Median birthweight was significantly higher among infants born at Mbekweni relative to those born at TC Newman. Overall, the median WAZ at birth was −0.55 (IQR: −1.31 to 0.07) and the median HCZ at birth was −0.54 (IQR: −1.29 to 0.15). WAZ and HCZ were significantly lower at TC Newman than at Mbekweni. Overall, the median HAZ at birth was 0.03 (IQR: −0.86 to 0.93); no site differences were observed for this birth outcome (Table 1).

### Correlates of Antenatal Exposure to Hazardous Alcohol Use

In unadjusted analyses, being of mixed ancestry, experiences of childhood trauma, recent experiences of IPV, a greater number of recent stressful life events, being above the threshold for probable depression, hazardous tobacco use, and hazardous drug use were associated with increased odds of hazardous alcohol use during pregnancy (Table 2). In contrast, women who had completed secondary school or had some tertiary education were less likely to report hazardous alcohol use relative to those with primary school education only (Table 2). In multivariable analyses, having completed secondary school (aOR = 0.48; 95% CI: 0.23, 0.98) remained associated with educed odds of hazardous alcohol use during pregnancy. Recent exposure to IPV (aOR = 2.08; 95% CI: 1.37, 3.18) and hazardous tobacco use (aOR = 5.03; 95% CI: 2.97, 8.52) also remained associated with enhanced odds of hazardous alcohol use during pregnancy (Table 2).

**Table 2:** Psychosocial Variables Associated with Antenatal Exposure to Hazardous Alcohol Use (*n* = 986)

Variable	No hazardous alcohol exposure (*n* = 855) *n* (%)	Hazardous alcohol exposure (*n* = 131) *n* (%)	Unadjusted association		*p*‐Value	Adjusted associations^a^		*p*‐Value
			Odds ratio	95% CI		Odds ratio	95% CI	
Ancestry								
African	488 (57.14)	41 (31.30)	Reference			Reference		
Mixed ancestry	366 (42.86)	90 (68.70)	2.93	(1.97; 4.34)	<0.001	0.95	(0.53; 1.70)	0.857
Educational attainment								
Primary	62 (7.25)	19 (14.50)	Reference			Reference		
Some secondary	448 (52.40)	80 (61.07)	0.58	(0.33; 1.02)	0.062	0.68	(0.36; 1.30)	0.246
Completed secondary	291 (34.04)	30 (22.90)	0.34	(0.18; 0.64)	0.001	0.48	(0.23; 0.98)	0.045
Any tertiary	54 (6.32)	2 (1.53)	0.12	(0.03; 0.54)	0.006	0.25	(0.05; 1.18)	0.080
Childhood trauma								
Above threshold	277 (32.40)	64 (48.85)	1.99	(1.38; 2.89)	<0.001	1.17	(0.75; 1.81)	0.491
Median number of stressful life events (IQR)	1 (0; 3)	2 (1; 4)	1.17	(1.09; 1.25)	<0.001	1.00	(0.92; 1.10)	0.949
Recent intimate partner violence								
Above threshold	257 (30.06)	76 (58.02)	3.22	(2.21; 4.69)	<0.001	2.08	(1.37; 3.18)	0.001
Depression								
Above threshold	178 (20.82)	49 (37.40)	2.27	(1.54; 3.36)	<0.001	1.52	(0.97; 2.38)	0.070
HIV‐seropositive	228 (26.60)	20 (15.27)	0.62	(0.37; 1.03)	0.064	0.93	(0.51; 1.70)	0.819
Hazardous tobacco use	193 (22.57)	86 (65.65)	6.56	(4.42; 9.73)	<0.001	5.03	(2.97; 8.52)	<0.001
Hazardous drug use	20 (2.34)	12 (9.16)	4.21	(2.01; 8.83)	<0.001	1.49	(0.66; 3.36)	0.342

CI, confidence interval.

a Model is adjusted for all other covariates in the table.

### Association Between Hazardous Alcohol Use and Birth Outcomes

First, we examined the association between hazardous alcohol use during pregnancy and infant WAZ at birth (Table 3). In unadjusted analyses, hazardous alcohol use during pregnancy was associated with significantly lower WAZ (*B* = −0.51, 95% CI: −0.69, −0.31). Other maternal predictors of WAZ included age, BMI, mixed ancestry, hazardous tobacco use, depression, childhood trauma, recent IPV, and number of stressful life events (Table 3). After adjusting for the potential confounding effect of these predictors in multiple regression analyses, hazardous alcohol use during pregnancy remained a significant predictor of infant WAZ (*B* = −0.35, 95% CI: −0.56, −0.14; Table 3).

**Table 3:** Association Between Antenatal Exposure to Hazardous Alcohol Use and Infant WAZ (*n* = 931), HAZ (*n* = 919), and HCAZ (*n* = 923) at Birth

Variable	Infant WAZ		Infant HAZ		Infant HCZ	
	Univariate regression coefficient (95% CI)	Multivariate regression coefficient (95% CI)^a^	Univariate regression coefficient (95% CI)	Multivariate regression coefficient (95% CI)	Univariate regression coefficient (95% CI)	Multivariate regression coefficient (95% CI)
Hazardous alcohol use	−0.51 (−0.69; −0.31)^b^	−0.35 (−0.56; −0.14)^b^	−0.65 (−0.92; −0.38)^b^	−0.46 (−0.76; −0.17)^b^	−0.54 (−0.78; −0.30)^b^	−0.43 (−0.69; −0.17)^b^
Maternal age	0.03 (0.01; 0.03)^b^	0.01 (−0.00; 0.02)	0.02 (0.00; 0.04)^b^	0.01 (−0.01; 0.02)	0.03 (0.02; 0.05)^b^	0.03 (0.02; 0.05)^b^
Maternal BMI	0.03 (0.02; 0.04)^b^	0.02 (0.01; 0.03)^b^	0.02 (0.00; 0.03)^b^	0.01 (−0.00; 0.03)	0.02 (0.01; 0.03)^b^	0.00 (−0.01; 0.02)
Parity						
0 to 1 child	Reference		Reference		Reference	
2 children	−0.14 (−0.34; 0.51)		0.06 (−0.21; 0.32)		−0.09 (−0.33; 0.16)	
3 children	0.09 (−0.21; 0.38)		0.26 (−0.15; 0.66)		0.06 (−0.31; 0.43)	
4 children	0.37 (−0.16; 0.87)		−0.16 (−0.82; 0.53)		0.55 (−0.06; 1.17)	
5 children	0.29 (−0.77; 1.34)		0.12 (−1.31; 1.54)		0.75 (−0.57; 2.07)	
6 children	−1.84 (−3.94; 0.26)		−1.27 (−4.11; 1.57)		−2.17 (−4.80; 0.46)	
Ancestry						
African	Reference	Reference	Reference	Reference	Reference	Reference
Mixed ancestry	−0.27 (−0.40; −0.13)^b^	−0.06 (−0.22; 0.11)	−0.22 (−0.40; −0.03)^b^	−0.00 (−0.22; 0.22)	−0.29 (−0.46; −0.13)^b^	−0.08 (−0.30; 0.12)
HIV‐sero‐positive	0.13 (−0.04; 0.29)		0.03 (−0.20; 0.25)		0.21 (−0.00; 0.41)	
Educational attainment						
Primary	Reference		Reference		Reference	
Some secondary	0.21 (−0.04; 0.46)		0.05 (−0.30; 0.39)		0.11 (−0.20; 0.41)	
Completed secondary	0.23 (−0.03; 0.49)		0.20 (−0.16; 0.56)		0.13 (−0.19; 0.45)	
Any tertiary	0.24 (−0.12; 0.61)		0.00 (−0.50; 0.50)		0.07 (−0.38; 0.52)	
Average household income						
<R1,000/month	Reference		Reference		Reference	Reference
R1,000 to R5,000/month	−0.10 (−0.25; 0.04)		−0.08 (−0.29; 0.11)		−0.17 (−0.34; −0.01)^b^	−0.21 (−0.39; −0.02)^b^
>R5,000/month	0.17 (−0.04; −0.38)		0.24 (−0.05; 0.52)		−0.09 (−0.17; 0.35)	0.03 (−0.24; 0.29)
Childhood trauma	−0.15 (−0.29; −0.00)^b^	−0.02 (−0.17; 0.13)	−0.21 (−0.40; −0.01)^b^	−0.13 (−0.34; 0.07)	−0.06 (−0.24; 0.11)	
Stressful life events	−0.15 (−0.29; −0.01)^b^	−0.01 (−0.04; 0.02)	−0.03 (−0.07; 0.02)		−0.06 (−0.09; −0.02)^b^	−0.02 (−0.06; 0.03)
Intimate partner violence	−0.18 (−0.32; −0.04)^b^	−0.06 (−0.21; 0.09)	−0.15 (−0.35; 0.04)		−0.20 (−0.37; −0.02)^b^	−0.02 (−0.20; 0.17)
Depression	−0.27 (−0.43; −0.11)^b^	−0.12 (−0.29; 0.06)	−0.22 (−0.43; 0.00)^b^	−0.08 (−0.31; 0.16)	−0.33 (−0.53; −0.13)^b^	−0.21 (−0.42; 0.00)
Hazardous tobacco use	−0.36 (−0.51; −0.22)^b^	−0.16 (−0.35; 0.03)	−0.35 (−0.55; −0.15)^b^	−0.21 (−0.47; 0.05)	−0.36 (−0.54; −0.18)^b^	−0.09 (−0.32; 0.15)
Hazardous drug use	−0.21 (−0.59; 0.17)		−0.14 (−0.66; 0.38)		−0.15 (−0.62; 0.32)	

CI, confidence interval.

a Adjusted for all variables significantly associated with outcome in univariate models.

b *p* < 0.05.

Next, we examined the relationship between hazardous alcohol use during pregnancy and infant HAZ (Table 3). In unadjusted analyses, infants born to mothers who drank at hazardous levels had significantly lower HAZ than infants without this exposure (*B* = −0.65, 95% CI: −0.92, −0.38). Other maternal variables that predicted HAZ included maternal age, maternal BMI, being of mixed ancestry, exposure to childhood trauma, depression, and hazardous tobacco use. After adjusting for the potential confounding effects of these other predictors in multiple regression analysis, hazardous alcohol use during pregnancy remained a significant predictor of infant HAZ (*B* = −0.46, 95% CI: −0.76, −0.17).

We also examined the effect of hazardous drinking during pregnancy on infant HCZ. In unadjusted analyses, infants with hazardous alcohol exposure had significantly lower HCZ than infants without this exposure (*B* = −0.54, 95% CI: −0.78, −0.30). Other variables that predicted infant HCZ included maternal age, maternal BMI, monthly household income of R1,000 to R5,000, hazardous tobacco use, maternal depression, recent IPV exposure, and number of stressful life events. After adjusting for the potential confounding effects of these variables in multiple regression analysis, hazardous alcohol use during pregnancy remained a significant predictor of infant HCZ (*B* = −0.43, 95% CI: −0.69, −0.17; Table 3).

## Discussion

This study examined associations of hazardous alcohol use, psychosocial stressors, and hazardous tobacco and illicit drug use with adverse birth outcomes in a cohort of pregnant South African women. Study findings build on those of previous cohort studies (e.g., Carter et al., 2012; Sania et al., 2017) by revealing the unique contribution that hazardous alcohol use makes to adverse birth outcomes after controlling for the potential confounding effects of maternal psychosocial stressors and other maternal risk behaviors. The main findings were that (i) a sizable proportion of women reported hazardous alcohol use during pregnancy; (ii) hazardous alcohol use was associated with infant growth restriction at birth, even after controlling for maternal BMI, psychosocial stressors, and hazardous tobacco and drug use; and (iii) hazardous tobacco use and experiences of IPV were associated with hazardous alcohol use during pregnancy.

In this study, almost 1 in 5 mothers of mixed ancestry reported hazardous drinking during pregnancy. This high rate of problem drinking highlights the urgent need for evidence‐based interventions to reduce hazardous alcohol use during pregnancy in this region. The finding that rates of hazardous alcohol use were significantly higher among mothers of mixed ancestry than African mothers is not surprising given historical differences in patterns of alcohol use and FASD rates between these communities (May et al., 2008; Parry et al., 2005). Nonetheless, we cannot rule out possible underreporting of hazardous alcohol use by African mothers. Other studies have noted that African women are less likely than women of mixed ancestry to disclose hazardous alcohol use to health providers due to cultural differences in the acceptability of alcohol use and stigma. Alcohol use is largely normative and culturally acceptable among women of mixed ancestry in this region, whereas it is traditionally only acceptable among African women under very tightly controlled circumstances (Myers et al., 2016). To ensure accurate screening of pregnant women for hazardous alcohol use, there is an urgent need for affordable and scalable technologies that are able to detect alcohol exposure accurately without the inherent limitations of self‐report measures (Muggli et al., 2015).

The need to introduce interventions to reduce hazardous alcohol use during pregnancy is underscored by findings that this antenatal exposure is associated with poorer infant growth outcomes, even after controlling for the confounding effects of maternal age, BMI, psychosocial stressors, and hazardous tobacco and illicit drug use. Even though we found relatively modest differences in birthweight, head circumference, and height for age among alcohol‐exposed and nonexposed infants, evidence from other studies suggests that alcohol‐related growth restrictions may persist into early childhood and could influence children's neurocognitive development (Carter et al., 2013, 2016). The DCHS is assessing the growth and neurodevelopmental outcomes of infants included in this cohort to determine the long‐term clinical significance of the magnitude of the alcohol‐related infant growth restrictions observed in this analysis. Given the possible long‐term consequences of alcohol‐related growth restrictions for child health, we recommend screening the mothers of infants who present with growth restrictions for hazardous alcohol use during pregnancy. This would allow for the early identification of infants with hazardous alcohol exposure who could benefit from therapeutic interventions aimed at enhancing their development.

In addition, findings provide guidance for the design of interventions to reduce hazardous alcohol use among pregnant women in South Africa. In keeping with findings reported by previous South African studies (Choi et al., 2014; Myers et al., 2015; O'Connor et al., 2011), mothers reporting hazardous tobacco use and past‐year exposure to IPV were more likely to report hazardous drinking during pregnancy than women without these exposures. This implies that interventions to reduce hazardous alcohol use during pregnancy should also address potential exposure to IPV and hazardous tobacco use. While the cross‐sectional nature of the psychosocial data precludes an understanding of the temporality of the relationship between IPV exposure and hazardous alcohol use, prior studies suggest that it is probably bidirectional. Earlier research found that women use alcohol to cope with experiences of IPV (Myers et al., 2016; Testa et al., 2003); this in turn increases their risk for future victimization (Wechsberg et al., 2013). Recent exposure to IPV may also lead to increased intake of alcohol once pregnant (O'Connor et al., 2011). Regardless of the direction of this relationship, the high rates of IPV exposure in this sample and evidence that trauma‐informed services are particularly efficacious for substance‐using women with histories of IPV (Raja et al., 2015) suggest that a combined approach to addressing trauma and alcohol problems among pregnant women is warranted.

In addition, there may be value in integrating alcohol interventions with smoking cessation programs for this population. In keeping with findings from high‐income countries (Oh et al., 2017), hazardous tobacco use was significantly associated with hazardous alcohol use in this sample. In contrast to previously published studies (Aliyu et al., 2009; Vanker et al., 2016), we found no evidence in support of the independent or synergistic effects (unreported analyses) of hazardous tobacco and alcohol use on fetal growth. The discrepancy between our findings and those from previous studies is probably due to differences in the assessment of maternal tobacco exposure. In particular, our use of self‐report dichotomous indicators rather than objective, quantitative measures of maternal tobacco use probably limited our ability to detect relationships between maternal tobacco use, alcohol use, and birth outcomes in adjusted models. Given this limitation and the well‐documented relationship between maternal tobacco use and birth outcomes (e.g., Castles et al., 1999), we argue that it is critical to screen all pregnant women for hazardous alcohol and tobacco use and to offer interventions to those women who screen positive for these exposures.

This study has some limitations common to other cohort studies. First, alcohol intake was measured by self‐reports, which are known to underestimate alcohol use among pregnant women (Muggli et al., 2015), largely due to social desirability and recall bias. Related to this, fieldworkers who administered the questionnaires were drawn from the community; this may have worsened social desirability bias although every effort was made to make women feel at ease. Second, we did not assess the volume or the gestational timing of alcohol consumption, both of which would have led to a more nuanced understanding of the relationship between alcohol exposure and birth outcomes. Third, as we only assessed exposure to psychosocial stressors at the second study visit, we were unable to account for any exposure to stressors that occurred later in pregnancy. Related to this, our psychosocial measures assessed a mix of past‐year, 3‐month and current exposure to stressors, and we therefore could not test whether these psychosocial exposures mediated the relationship between hazardous alcohol use and fetal growth restrictions. Greater consideration should be given to the choice and timing of these assessments in future studies so that potential mediators and moderators of the relationship between hazardous alcohol use and fetal outcomes can be examined. Further, the clinics in our sample serve pregnant women from 1 health subdistrict in the Western Cape and are not necessarily similar to other clinics in the province; thus, the extent to which findings are generalizable to other subdistricts is unclear. The mothers in our sample also may not be representative of all pregnant women who drink in this region, many of whom do not seek antenatal care until very late in their pregnancy (Williams et al., 2014). Despite these limitations, our findings are largely in keeping with other community‐recruited samples of women who reported drinking during pregnancy (Choi et al., 2014; O'Connor et al., 2011; Sania et al., 2017), increasing our confidence in the validity of these results.

## Conclusions

Despite some limitations, study findings have implications for the development of health services aimed at promoting maternal and infant well‐being. First, findings of high levels of hazardous alcohol use point to the need for ongoing community‐based education around the risks associated with hazardous alcohol use during pregnancy. Second, findings suggest that universal screening of pregnant women for hazardous alcohol use (as well as tobacco use and exposure to IPV) is required to identify and link pregnant women with these risks to appropriate interventions. Third, findings indicate that alcohol‐reduction interventions should target not only hazardous alcohol use but also potential exposure to IPV and co‐occurring tobacco use. Given the exceptionally high rates of IPV and other forms of trauma in South African society (Choi et al., 2014), trauma‐informed interventions that address alcohol and tobacco use in an integrated manner may be particularly effective for reducing hazardous alcohol use among pregnant women in South Africa. Randomized controlled trials of trauma‐informed interventions for maternal alcohol use must still confirm this hypothesis. Fourth, our findings of alcohol‐associated infant growth restrictions have implications for the early identification of infants with potential exposure to hazardous alcohol use. We recommend retrospective screening of all infants with growth restrictions for hazardous alcohol exposure and their linkage, if required, to early childhood development programs to address potential developmental problems.

## Funding

This study was funded by the Bill and Melinda Gates Foundation (OPP 1017641). BM, DJS, HJZ, NK, and WB are supported by the South African Medical Research Council.

## Conflict of Interest

The authors have no conflict of interest to declare.
